# PTPIP51 regulates mouse cardiac ischemia/reperfusion through mediating the mitochondria-SR junction

**DOI:** 10.1038/srep45379

**Published:** 2017-03-27

**Authors:** Xue Qiao, Shi Jia, Jingjing Ye, Xuan Fang, Chenglin Zhang, Yangpo Cao, Chunling Xu, Lifang Zhao, Yi Zhu, Lu Wang, Ming Zheng

**Affiliations:** 1 Department of Physiology and Pathophysiology, School of Basic Medical Sciences, Health Science Center, Peking University, Beijing 100191, China; 2 Center for Human Disease Genomics, School of Basic Medical Sciences, Health Science Center, Peking University, Beijing 100191, China

## Abstract

Protein tyrosine phosphatase interacting protein 51 (PTPIP51) participates in multiple cellular processes, and dysfunction of PTPIP51 is implicated in diseases such as cancer and neurodegenerative disorders. However, there is no functional evidence showing the physiological or pathological roles of PTPIP51 in the heart. We have therefore investigated the role and mechanisms of PTPIP51 in regulating cardiac function. We found that PTPIP51 was markedly upregulated in ischemia/reperfusion heart. Upregulation of PTPIP51 by adenovirus-mediated overexpression markedly increased the contact of mitochondria-sarcoplasmic reticulum (SR), elevated mitochondrial Ca^2+^ uptake from SR release through mitochondrial Ca^2+^uniporter. Inhibition or knockdown of mitochondrial Ca^2+^uniporter reversed PTPIP51-mediated increase of mitochondrial Ca^2+^ and protected cardiomyocytes against PTPIP51-mediated apoptosis. More importantly, cardiac specific knockdown of PTPIP51 largely reduced myocardium infarction size and heart injury after ischemia/reperfusion. Our study defines a novel and essential function of PTPIP51 in the cardiac ischemia/reperfusion process by mediating mitochondria-SR contact. Downregulation of PTPIP51 improves heart function after ischemia/reperfusion injury, suggesting PTPIP51 as a therapeutic target for ischemic heart diseases.

Cardiac ischemia/reperfusion (I/R) injury usually accompanies coronary heart disease, which is one of the leading causes of heart failure^1^,^2^. A variety of mechanisms including I/R-triggered excess reactive oxygen species (ROS) production, intracellular calcium overload, dysfunction of cardiac contractile activity, and necrotic or apoptotic cell death, have been proposed to explain cardiac dysfunction and subsequently pathological processes after cardiac I/R^3^,^4^,^5^. Mitochondria, the energy-supplying organelles, are important components of the intracellular calcium buffering system and the primary resource of ROS production, and thus they are central determinants of cell survival and death and are gradually recognized as pivotal players in the genesis of I/R injury^3^,^6^. As a metabolically active tissue, heart cells contain prominent mitochondrial networks, occupying up to 40% of cell volume and generating ATP in response to energy needs through oxidative phosphorylation (OXPHOS)^7^,^8^. Dysfunction of mitochondria, such as decreased mitochondrial electron transport chain activity and mitochondrial ATP synthesis, increased ROS production, the irreversible opening of the mitochondrial permeability transition pore (mPTP), release of cytochrome C from mitochondria to cytosol, and eventually cell death, are closely associated with cardiac I/R^9^,^10^,^11^. Inhibition of mitochondrial complex I by rotenone, targeting overexpression of catalase in mitochondria, modulation of mitochondrial dynamics by inhibiting mitochondrial fission, or inhibition of mPTP by cyclosporine A, all significantly protected heart against I/R injury^12^, further suggesting a crucial role of mitochondria in cardiac I/R injury.

Mitochondria and the endoplasmic reticulum (ER) or the sarcoplasmic reticulum (SR in cardiomyocytes) are organelles physically associated through mitochondria-associated ER membranes (MAMs)^13^. The Ca^2+^ microdomains between mitochondria and SR in cardiomyocytes are tightly regulated by the mitochondria-SR interaction site^14^,^15^. During cardiac I/R, a rise in intracellular Ca^2+^ has been suggested to play an important role in the genesis of cardiac injury^16^. In addition to modulating Ca^2+^-dependent signals, the increased cytosolic Ca^2+^ subsequently leads to mitochondrial Ca^2+^ overload, which in turn triggers mPTP opening and eventually results in cell death^3^. The mitochondria-ER interaction site has been implicated in cytosolic Ca^2+^ accumulation induced mitochondrial Ca^2+^ overload^14^,^17^. Several proteins, such as VDAC1, Grp75, IP3R1, and more recently, mitofusin 2, have been identified to localize at the mitochondria-ER interaction site, regulating Ca^2+^ transfer from ER to mitochondria in different cell types^18^,^19^,^20^. In the heart, I/R induces increased interaction of cyclophilin D with MAMs macrocomplex VDAC1/Grp75/IP3R1 and leads to mitochondrial Ca^2+^ overload. Disruption of the mitochondria-SR interaction by either inhibition of CypD/VDAC1/Grp75/IP3R1 or downregulation of mitofusin2, attenuates mitochondrial Ca^2+^ overload and protects cardiomyocytes against I/R injury^21^.

Protein tyrosine phosphatase interacting protein 51 (PTPIP51) is widely expressed in mammalian tissues including the heart^22^. Our previous study in cell lines found that PTPIP51 primarily localizes on the mitochondrial surface through its N-terminal putative transmembrane motif^23^. In CV1 and NSC34 motor neuron cells, the amyotrophic lateral sclerosis (ALS)-related vesicle-associated membrane protein-associated protein-B (VAPB) has been found to interact with PTPIP51 at the mitochondria-ER junction; downregulation of both proteins impairs mitochondrial Ca^2+^ uptake and the mutated VAPB-P56S is enriched in MAMs fraction in ALS patients and causes mitochondrial transportation disruption and Ca^2+^ overload^24^,^25^,^26^. In the present study, we investigated the possible involvement of PTPIP51 in cardiac I/R-induced mitochondrial Ca^2+^ overload and cell death. We found that I/R upregulated PTPIP51 protein, which subsequently induced mitochondrial Ca^2+^ overload and cardiomyocyte death by enhancing Ca^2+^ transfer from SR to mitochondria through increasing mitochondria-SR contact sites. Downregulation of PTPIP51 in the heart protected I/R-induced cardiac dysfunction and cell death. Thus, our study reveals a novel role of PTPIP51 in regulating the mitochondria-SR junction and in cardiac function, and provides a new therapeutic target for cardiac I/R-related diseases.

## Results

### PTPIP51 is upregulated in mouse I/R hearts

**Figure 1:**
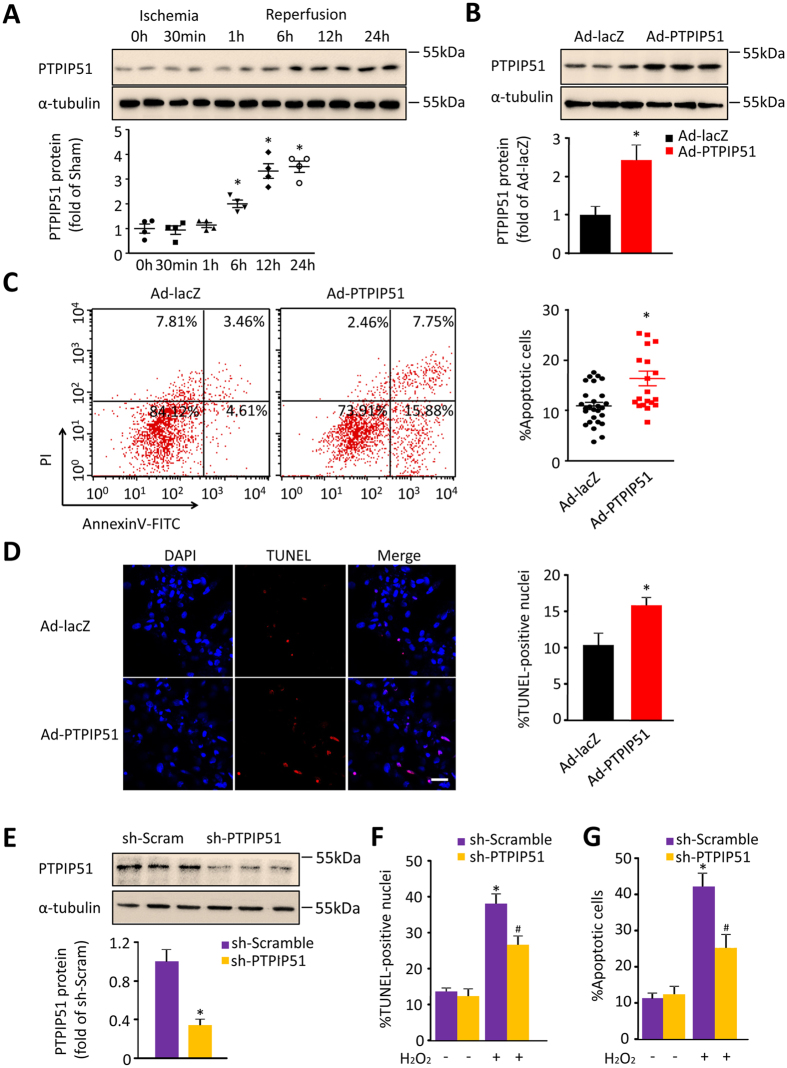
PTPIP51 was increased in mouse I/R hearts. (**A**) Western blot and scatter dot plots of PTPIP51 protein in mouse hearts from Sham and I/R border area. n = 4 independent experiments. α-tubulin was used as a protein loading control. *P < 0.05versus sham control group. (**B**) Western blot of adenoviral-mediated PTPIP51 expression (Ad-PTPIP51) in rat neonatal cardiomyocytes. Cardiomyocytes infected with adenovirus vector (Ad-lacZ) were used as a control. n = 3 independent experiments. (**C**) Annexin V-PI assay by flow cytometry and scatter dot plots, and (**D**) TUNEL staining, in Ad-lacZ and Ad-PTPIP51-infected cardiomyocytes. n = 27 (lacZ) and 20 (PTPIP51) experiments for (**C**), and n = 8–10 frames in each group for (**D**). Scale bar: 25 μm. *P < 0.05versus Ad-lacZ group. (**E**) PTPIP51 protein in rat neonatal cardiomyocytes infected with adenovirus-mediated PTPIP51 shRNA (sh-PTPIP51) or scrambled shRNA (sh-Scramble) as a control. n = 3 independent experiments. (**F**) TUNEL staining, and (**G**) Annexin V-PI assay, in sh-Scramble and sh-PTPIP51-infected rat neonatal cardiomyocytes. Cells were treated with 200 μM H_2_O_2_ for 12 h or with ddH_2_O as a control. n = 10–18 frames in each group for (**F**) and n = 5–8 independent experiments for (**G**). *P < 0.05 versus scramble group; ^*#*^*P* < 0.05 versus scramble group with H_2_O_2_ treatment.

PTPIP51 is expressed ubiquitously in many tissues including the heart, however, its cardiac function has not yet been studied. To investigate the cardiac function of PTPIP51 and its pathophysiological relevance, we examined the expression level of PTPIP51 in I/R heart. Mouse cardiac I/R models were set up with 30min ischemia followed by 1, 6, 12, 24-h reperfusion, and cardiac function was confirmed by echo assay (Fig. S1A and S1B). PTPIP51 protein levels at zones bordering the I/R site increased to 2.0-, 3.0-, 3.5-fold of the sham hearts at 6, 12, and 24-h after I/R (Fig. 1A), suggesting a possible involvement of PTPIP51 in the process of cardiac I/R injury.

We then investigated how elevated PTPIP51 participates in the pathogenesis of cardiac I/R. To answer this question, we employed both gain-of-function and loss-of-function approaches. Overexpressing PTPIP51 in neonatal rat cardiomyocytes by adenovirus-mediated gene transfer led to a 2.4 ± 0.4-fold increase in PTPIP51 protein abundance (Fig. 1B), parallel to that in I/R heart (Fig. 1A). Upregulated PTPIP51 resulted in increased apoptotic cardiomyocytes to 16.26 ± 1.46%, compared with 10.85 ± 0.72% of control cells, as indicated by flow cytometry analysis using fluorescence-labeled Annexin V to detect the translocation of phosphatidylserine (PS) from the cytoplasmic to the external cell membrane (Fig. 1C), a key biochemical marker of apoptotic cell death^23^. The apoptotic effect of PTPIP51 was further confirmed by TUNEL assay, with an apoptotic rate of 15.88 ± 1.0% in PTPIP51 overexpressing cardiomyocytes and 10.44 ± 1.65% in control cells (Fig. 1D). Together, these data show that upregulated PTPIP51 is sufficient to cause cardiomyocyte apoptosis.

We then determined if PTPIP51 is required for H_2_O_2_-induced cardiomyocyte apoptosis using the loss-of-function approach. PTPIP51 protein was knocked down by adenovirus-mediated PTPIP51 shRNA (adeno-PTPIP51 shRNA) in cardiomyocytes, to approximately one-third (31.3 ± 0.41%) of the level compared with scramble (adeno-scramble) control cells (Fig. 1E). Concomitantly, while the apoptotic cell rates showed no difference between adeno-scramble cells and adeno-PTPIP51 shRNA cells at the basal level, PTPIP51 knockdown reduced the apoptotic rate in response to H_2_O_2_ stimulation (200 μM, 12 h) from 38.39 ± 2.66% in the control cells to 25.54 ± 3.65% in adeno-PTPIP51 shRNA cells, as indicated by TUNEL staining assay (Fig. 1F and Fig. S1C). Annexin V-PI staining showed similar results: PTPIP51 knockdown largely decreased H_2_O_2_-induced apoptotic cardiomyocytes as compared with adeno-scramble control cells (Fig. 1G and Fig. S1D). Thus, the results show that PTPIP51 is required for H_2_O_2_-mediated cardiomyocyte apoptosis. Together, our data suggest that the elevation of PTPIP51 may contribute to I/R-induced cardiac injury.

### Knockdown of PTPIP51 in heart protects cardiac function after I/R

**Figure 2:**
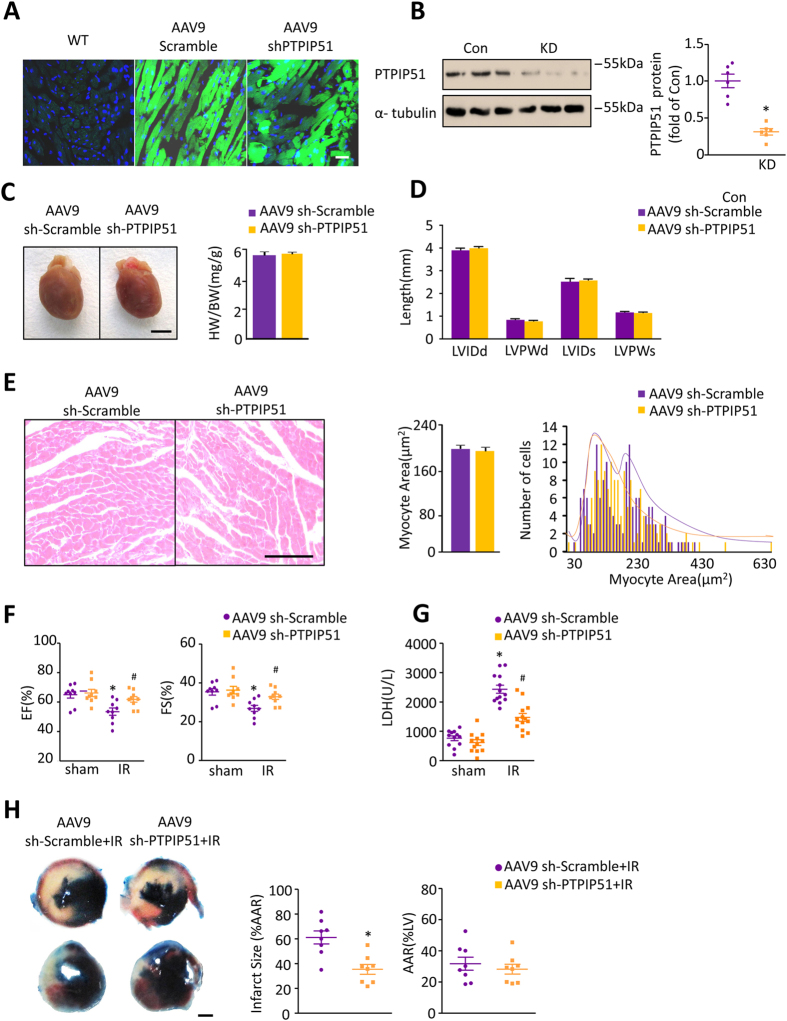
PTPIP51 knockdown protected mouse cardiac function after I/R. (**A**) Confocal images of AAV9-ZsGreen-scramble or AAV9-ZsGreen-PTPIP51 shRNA adenovirus transfection efficiency in mouse left ventricle. Scale bar: 25 μm. (**B**) PTPIP51 protein levels. n = 6 pairs of mice. *P < 0.05 versus Control(Con) group. (**C**) AAV9-sh-Scramble and AAV9-sh-PTPIP51 hearts. Bar chart is heart weight/body weight (HW/BW) ratio. (**D**) Bar chart is the LVID (left ventricular internal diameter) and LVPW (left ventricular posterior wall) length, d = in diastole, s = in systole. n = 9 pairs of mice. (**E**) HE staining of heart tissues from AAV9-sh-Scramble and AAV9-sh-PTPIP51 mice and cardiomyocyte cross-sectional area and frequency distribution were calculated. n = 150 cells from four pairs of mice. (**F**) Ejection fraction (EF, left) and fractional shortening (FS, right) by echocardiography of sh-Scramble and sh-PTPIP51 mouse hearts with or without I/R. n = 9 pairs of mice. (**G**), Serum LDH levels in sh-Scramble or sh-PTPIP51 mouse hearts with or without I/R. n = 12 pairs of mice. *P < 0.05 versus sham scramble; ^*#*^*P* < 0.05versus scramble with I/R injury. (**H**) Infarct sizes by triphenyltetrazolium chloride (TTC)-Evan’s Blue staining. Average data showing the percentages of infarct area to AAR (area at risk) and AAR to LV (left ventricular). n = 8 pairs of mice.

To further understand the *in vivo* function of PTPIP51 in the heart, we employed an adenovirus associated virus (AAV)-mediated knockdown approach in mice^27^,^28^,^29^ (Fig. S2A). Four weeks after the mouse was injected with PTPIP51 shRNA containing AAV9-ZsGreen (AAV9-ZsGreen-shPTPIP51), over 60% of cardiomyocytes were transfected with AAV9-ZsGreen-shPTPIP51 adenovirus, as visualized by green fluorescence signals (Fig. 2A), and PTPIP51 protein was reduced to 38% of scramble control hearts (Fig. 2B). There were no differences in heart weight/body weight ratio (Fig. 2C), ventricle size (Fig. 2D), and cardiomyocyte area (Fig. 2E) between PTPIP51 knockdown and scramble control mice.

We then tested the *in vivo* effect of PTPIP51 knockdown in heart I/R injury. Compared with scramble control hearts, echocardiography showed that knockdown of PTPIP51 did not change basic cardiac function (Fig. 2F& Fig. S2B). However, PTPIP51 knockdown mice displayed surprisingly improved cardiac function after 30 min ischemia followed by 24 h reperfusion, with a 12.4% higher fractional shortening and a 20.3% higher ejection fraction than the control hearts (Fig. 2F& Fig. S2B). Consistently, the infarction size (% area at risk) in PTPIP51 knockdown hearts after I/R was significantly smaller than the control hearts, while the areas at risk (% left ventricular) showed no significant difference between the two groups, based on Evan’s Blue-TTC double staining (Fig. 2H). After I/R, the serum lactic dehydrogenase (LDH) level, a biomarker of injury myocardium, increased from 763.6 ± 80.32 U/L to 2435 ± 135.7 U/L in scramble control mice (Fig. 2G). However, the serum LDH level in PTPIP51 knockdown mice was only 60.57% of that in control mice (Fig. 2G), suggesting a strong protective effect to I/R of PTPIP51 knockdown in the heart. Moreover, PTPIP51 knockdown protected cardiomyocytes from I/R injury-induced cardiomyocyte apoptosis, as shown by the decreased TUNEL staining positive cells in the border zone of PTPIP51 knockdown hearts after I/R injury comparing with scramble control hearts (Fig. S2C). Together, the *in vivo* results provide solid evidence supporting the pivotal role of PTPIP51 in cardiac I/R injury, and more importantly, suggesting a protective strategy against cardiac I/R injury by decreasing the PTPIP51 level.

### PTPIP51 regulates mitochondrial and cytosolic Ca^2+^ in cardiomyocytes

**Figure 3:**
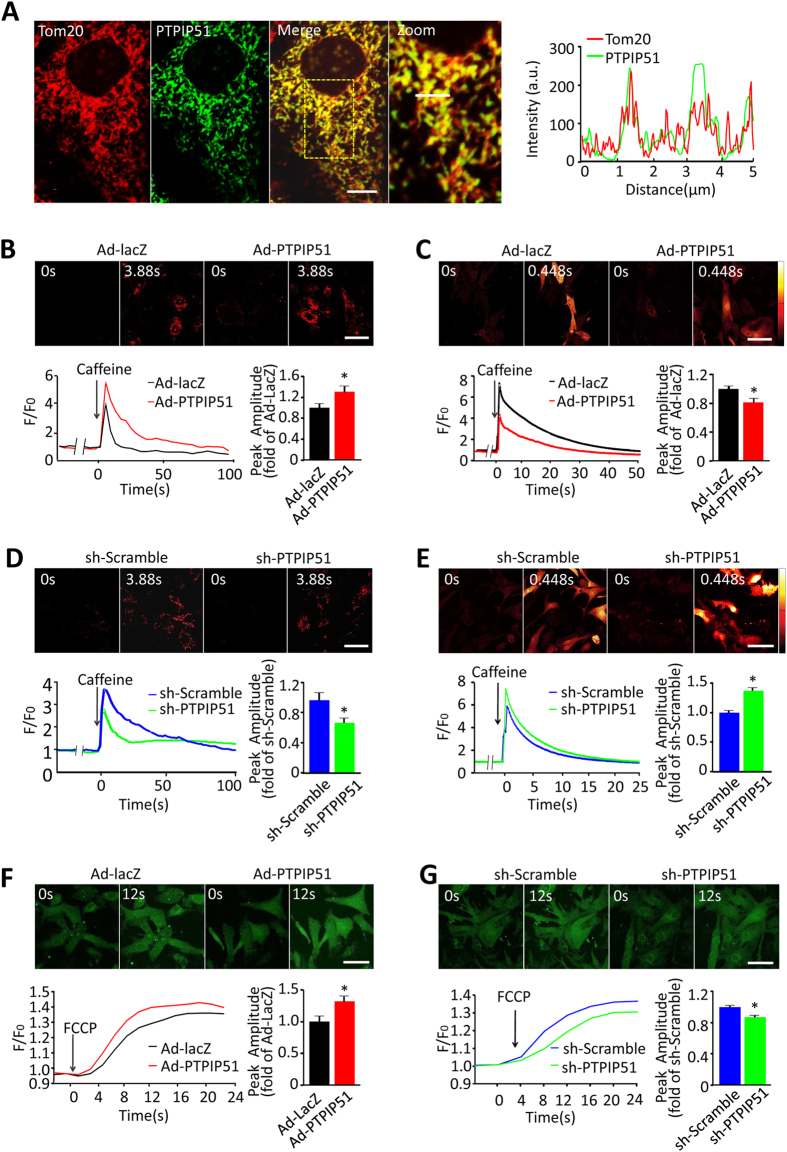
PTPIP51 increased mitochondrial and decreased cytosolic Ca^2+^ in cardiomyocytes. (**A**) Confocal images of rat neonatal cardiomyocytes immunostained with a rabbit-anti-PTPIP51 antibody and mouse-anti-Tom20, and distribution curves of immunofluorescent intensities (right). Scale bar: 10 μm in merge, and 5 μm in Zoom. (**B**) Confocal images and representative recordings of mitochondrial Ca^2+^ in rhod-2 AM-loaded neonatal cardiomyocytes transfected with Ad-PTPIP51 or Ad-lacZ in response to 10 μM caffeine stimulation. n = 41–42 cells in each group from five independent experiments. (**C**) Confocal images and representative recordings of cytosolic Ca^2+^ by Fluo-4 AM fluorescent signal in response to 10 μM caffeine stimulation, in cardiomyocytes transfected with Ad-PTPIP51 or Ad-lacZ. n = 29–31 cells in each group from five independent experiments. *P < 0.05versus Ad-lacZ group. (**D**) Confocal images and representative recordings of mitochondrial Ca^2+^ and (**E**) Cytosolic Ca^2+^ in response to 10 μM caffeine stimulation in neonatal cardiomyocytes transfected with PTPIP51 shRNA or scramble shRNA. n = 30–50 cells in each group from three independent experiments in (**D**) and n = 63–90 cells in each group from five independent experiments in (**E**). *P < 0.05 versus scramble group. (**F** & **G**) Confocal images and representative recordings of cytosolic Ca^2+^ in response to 1 μM FCCP stimulation in neonatal cardiomyocytes transfected with Ad-PTPIP51 (**F**) or with PTPIP51 shRNA (**G**). Data are presented as mean ± SEM. Bar charts show quantifications of peak amplitude.

To understand the underlying mechanisms of PTPIP51-mediated cardiac function, we at first targeted the localization of PTPIP51 in cardiomyocytes by immunofluorescent staining. The fluorescent signals from the PTPIP51 antibody and mitochondrial outer membrane protein Tom20 antibody overlapped, suggesting PTPIP51 mainly localizes on mitochondria in rat neonatal cardiomyocytes (Fig. 3A). This was in general agreement with previous studies that were carried out in CV1 and NSC34 motor neuron cells^24^,^30^. Because mitochondrial calcium signals have an important role in cell apoptosis^31^,^32^, we then examined the possible involvement of mitochondrial calcium in PTPIP51-mediated cardiomyocyte apoptosis. In neonatal rat cardiomyocytes loaded with mitochondrial Ca^2+^ probe Rhod-2 AM, SR Ca^2+^ release by caffeine stimulation induced a higher mitochondrial calcium signal in PTPIP51-overexpressing cells than in control cells, as indicated by the 1.3-fold increase in peak amplitude over the control cells (Fig. 3B). In contrast to the increased mitochondrial Ca^2+^ signal, cytosolic Ca^2+^ peak amplitude in PTPIP51 cardiomyocytes was only 80.2 ± 6.9% of that in control cells, indicated by the fluorescent intensity of calcium probe Fluo-4 AM (Fig. 3C). The contrasting changes in mitochondrial and cytosolic Ca^2+^ in response to SR Ca^2+^ release in PTPIP51 overexpressing cardiomyocytes indicate that PTPIP51 increases mitochondrial Ca^2+^ uptake during SR Ca^2+^ release. The role of PTPIP51 in regulating the mitochondrial Ca^2+^ signal was also checked in cardiomyocytes transfected with adeno-PTPIP51 shRNA. Knockdown of PTPIP51 caused a 31.7% decrease of mitochondrial Ca^2+^ signal and 1.3-fold increase of cytosolic Ca^2+^ signal in response to caffeine stimulation, compared with adeno-scramble cells, indicated by Rhod-2 AM and Fluo-4 AM fluorescence respectively (Fig. 3D and E). Together, these results from both gain-of-function and loss-of-function studies reveal that in cardiomyocytes, PTPIP51 elevates mitochondrial Ca^2+^ uptake in response to SR Ca^2+^ release.

Furthermore, we checked mitochondrial Ca^2+^ content by treating resting cardiomyocytes with carbonyl cyanide 4-(trifluoromethoxy) phenylhydrazone (FCCP), the potent mitochondrial oxidative phosphorylation uncoupler, to disrupt the mitochondrial membrane potential and release mitochondrial Ca^2+^ to the cytosol. We found that cytosolic Ca^2+^ increased more in PTPIP51-overexpressing cells, to a 1.3-fold of that in control cells, as indicated by the increased Fluo-4 AM fluorescent intensity, suggesting more mitochondrial Ca^2+^ release to the cytosol (Fig. 3F). Whereas, FCCP stimulation caused a 14% decreased mitochondrial Ca^2+^ release into the cytosol compared with that in control cells (Fig. 3G). Collectively, our results support the regulatory role of PTPIP51 on mitochondrial Ca^2+^ content in the resting state and mitochondrial Ca^2+^ uptake in response to SR Ca^2+^ releases in cardiomyocytes.

### PTPIP51 increases mitochondrial calcium through mitochondrial calcium uniporter

**Figure 4:**
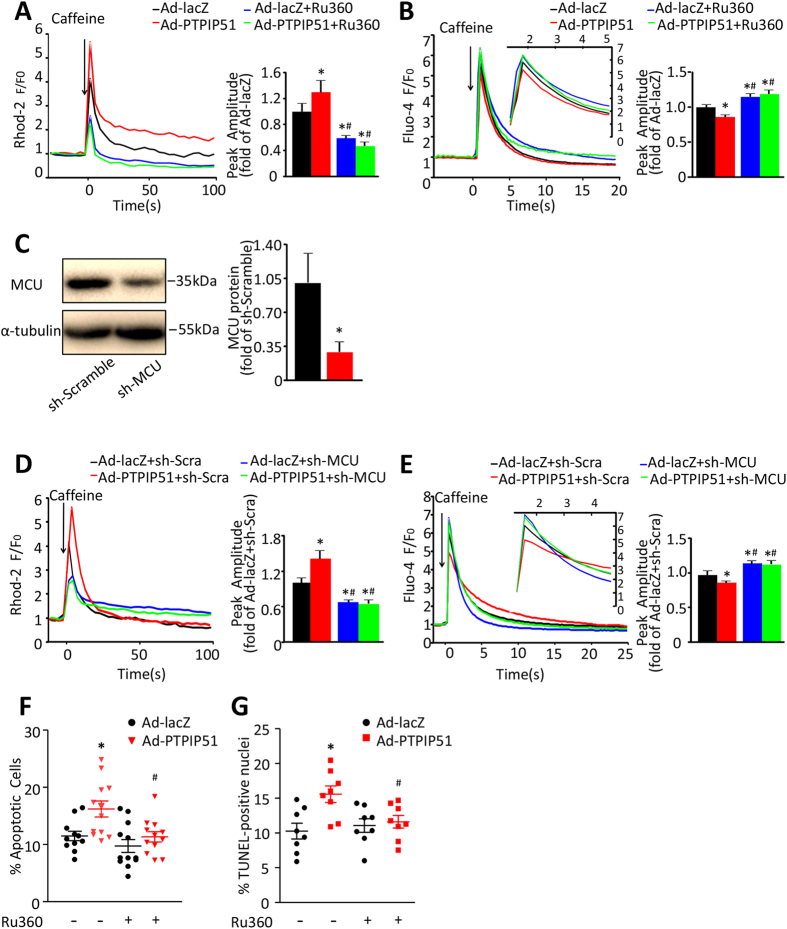
PTPIP51 regulated cell function through increasing mitochondrial calcium influx. (**A**) Mitochondrial Ca^2+^ and (**B**) Cytosolic Ca^2+^ recordings of neonatal cardiomyocytes transfected with Ad-lacZ or Ad-PTPIP51 in the presence or absence of MCU inhibitor Ru360 (2 μM, for 30 min), in response to caffeine stimulation. n = 41–70 cells in each group for (**A**) and n = 46–100 cells in each group for (**B**) from five independent experiments. Bar charts show quantifications of peak amplitude. *P < 0.05versus Ad-lacZ; ^*#*^*P* < 0.05 versus Ad-PTPIP51. (**C**) MCU protein level in sh-MCU-transfected cardiomyocytes. n = 3 independent experiments. *P < 0.05 versus sh-Scramble. (**D**) Mitochondrial Ca^2+^ and (**E**) Cytosolic Ca^2+^ recordings of neonatal cardiomyocytes transfected with Ad-lacZ, or Ad-PTPIP51 and co-transfected with sh-scramble (sh-Scra) or sh-MCU, in response to caffeine stimulation. n = 35–64 in (**D**) and n = 54–82 in (**E**) in each group from five independent experiments. Bar charts show quantifications of peak amplitude. *P < 0.05 versus Ad-lacZ + sh-scramble; ^*#*^*P* < 0.05 versus Ad-PTPIP51 + sh-scramble. (**F**) Annexin V-PI assay by flow cytometry and (**G**) TUNEL staining in cardiomyocytes transfected with Ad-lacZ or Ad-PTPIP51 with/without Ru360. n = 12 (**F**) and n = 8 (**G**) independent experiments. *P < 0.05versus Ad-lacZ; ^*#*^*P* < 0.05 versus Ad-PTPIP51.

Mitochondrial calcium uniporter (MCU) is an inward rectifying, low-affinity, and high-capacity channel that regulates Ca^2+^ flux into the mitochondrial matrix^33^,^34^. To understand if MCU is required for PTPIP51-mediated mitochondrial Ca^2+^ increase, we treated PTPIP51-overexpressing cardiomyocytes with Ru360, a specific inhibitor, to block MCU activity. Ru360 decreased the peak amplitude of mitochondrial Ca^2+^ in response to caffeine stimulation in PTPIP51-overexpressing cardiomyocytes to 36.1% of that in cells without Ru360 treatment, with a similar level as in lacZ control cells treated with Ru360 (Fig. 4A). Consistently, cytosolic Ca^2+^ peak amplitude (as shown by Fluo-4 fluorescent signal), in response to caffeine stimulation in PTPIP51-overexpressing cardiomyocytes in the presence of Ru360, was increased by 1.4-fold compared with the same cells without Ru360 treatment (Fig. 4B). We further confirmed the role of MCU in PTPIP51-mediated mitochondrial Ca^2+^ flux by transfecting cardiomyocytes with adenovirus-mediated MCU shRNA (adeno-MCU shRNA). MCU protein was knocked down to approximately one third of that in scramble (adeno-scramble) control cells (Fig. 4C). Similar to Ru360 treatment, adeno-MCU shRNA co-transfection in PTPIP51-overexpressing cardiomyocytes led to decreased mitochondrial Ca^2+^ amplitude and increased cytosolic Ca^2+^ amplitude in response to caffeine stimulation, as compared with adeno-scramble/PTPIP51 overexpressing cardiomyocytes (Fig. 4D and E). Thus, these results suggest that PTPIP51 mediates mitochondrial Ca^2+^ flux through mitochondrial Ca^2+^ uniporter MCU.

Furthermore, inhibition of MCU by Ru360 protected PTPIP51-mediated cardiomyocyte apoptosis, as shown by Annexin V-PI assay (Fig. 4F) and TUNEL staining (Fig. 4G). Collectively, our data suggest that PTPIP51 causes mitochondrial Ca^2+^ overload through MCU, which subsequently leads to cardiomyocyte death.

### PTPIP51 increases cardiomyocyte mitochondria-SR contacts

**Figure 5:**
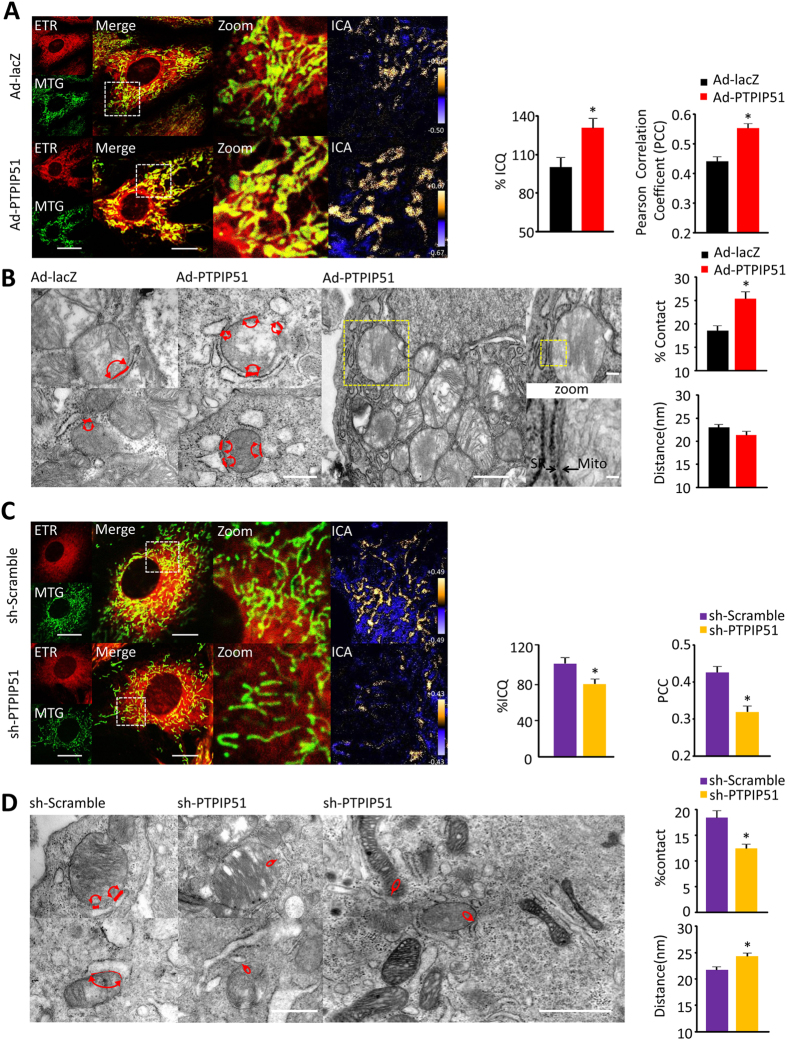
PTPIP51 increased mitochondria-SR contacts in cardiomyocytes. (**A**) Confocal images of Ad-lacZ or Ad-PTPIP51 transfected cardiomyocytes loaded with Mito-Tracker Green and ER-Tracker Red. Bar charts are mean intensity correlation quotients (ICQ) (left) and Pearson correlation coefficient (PCC) (right). n = 59–61 frames in each group from three independent experiments. Scale bars: 7 μm and 3.5 μm (Merge). Green: Mito-Tracker green; Red: ER-Tracker red; ICA: intensity correlation of green and red fluorescent signals and high correlation is displayed in yellow. (**B**) TEM images of mitochondria-SR contacts in neonatal cardiomyocytes as indicated. Red lines with arrows indicate mitochondria-SR contacts. Scale bar: 500 nm. Yellow square areas in the Ad-PTPIP51 cell were further zoomed in to show the tight contacts of mitochondria (Mito) and SR (arrowheads). Scale bars: 1 μm, 200 nm (zoom upper), and 40 nm (zoom bottom). Bar charts are % of the mitochondrial surface closely in contact with SR (upper) and mean distance between mitochondria and SR junction (bottom). n = 37–50 frames in each group from four independent experiments. *P < 0.05 versus Ad-lacZ. (**C**) Confocal images of scramble or PTPIP51 shRNA-transfected cardiomyocytes loaded with Mito-Tracker Green and ER-Tracker Red. Bar charts are mean ICQ (left) and PCC (right). n = 69 frames in each group from three independent experiments. Scale bars: 7 μm and 3.5 μm (Merge). (**D**) TEM images of mitochondria-SR contacts in neonatal cardiomyocytes as indicated. Red lines are mitochondria-SR contacts. Scale bar: 500 nm. Low-magnification TEM image of PTPIP51 shRNA-transfected cardiomyocytes (right). Scale bar: 1 μm. Bar charts are % of the mitochondrial surface closely in contact with SR (upper) and mean distance between mitochondria and SR junction (bottom). n = 43–45 frames in each group from four independent experiments. *P < 0.05 versus scramble group.

Mitochondria and ER/SR are closely associated organelles in most cell types including cardiomyocytes, and the contact sites of mitochondria-ER/SR are involved in the regulation of intracellular Ca^2+^ through controlling mitochondrial Ca^2+^ buffering function^13^,^35^,^36^. Based on our findings that PTPIP51 increased mitochondrial Ca^2+^ flux through MCU while decreasing cytosolic Ca^2+^ during SR calcium release, we hypothesized that PTPIP51 regulates the mitochondrial Ca^2+^ signal through changing mitochondria-SR junction sites. In either PTPIP51-overexpressing or knockdown cardiomyocytes, mitochondria and SR were visualized by fluorescent signals of MitoTracker Green and ERTracker Red, respectively, and the overlapping of the green fluorescent signal from MitoTracker and the red fluorescent signal from ER Tracker was quantified by intensity correlation quotient (ICQ) and analyzed by Pearson correlation coefficient^30^,^37^. Interestingly, overexpression of PTPIP51 caused a 30.6 ± 7.7% increase in ICQ compared with control cardiomyocytes, and the Pearson correlation coefficient increased from 0.44 in control to 0.55 in PTPIP51 expressing cardiomyocytes (Fig. 5A). Electron microscopy data showed that the contact ratio of the mitochondria-SR membrane was largely increased by PTPIP51 overexpression, to about 136.8% of that in control cells, although the distance between the two organelles showed no difference (Fig. 5B). In contrast to the increased mitochondria-SR contact ratio by PTPIP51 overexpression, knockdown of PTPIP51 by adeno-PTPIP51 shRNA led to a 21.5% reduction of ICQ compared with control and Pearson correlation coefficient decreased from 0.43 in control to 0.32 in PTPIP51 knockdown cardiomyocytes (Fig. 5C). Similarly, electron microscopy analysis showed a 32.6% decrease in the mitochondria-SR contact ratio in PTPIP51 knockdown cardiomyocytes compared with scramble control cells. Surprisingly, PTPIP51 knockdown also increased the distance between mitochondria and SR, from about 20 nm in scramble control cardiomyocytes to about 25 nm in PTPIP51 knockdown cells (Fig. 5D). These results support our hypothesis that PTPIP51 regulates mitochondria-SR junction sites in cardiomyotyes.

**Figure 6:**
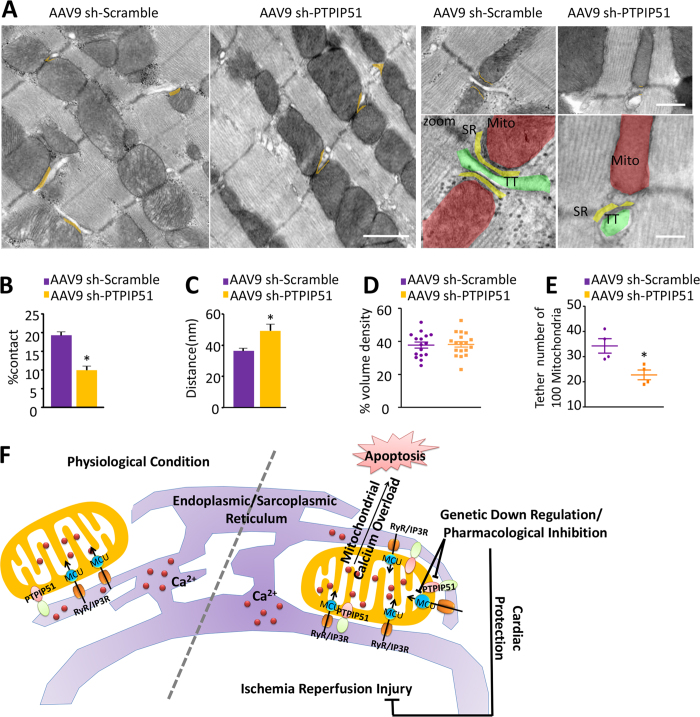
PTPIP51 knockdown decreased mitochondria-SR contacts in mouse hearts. (**A**) TEM images showing mitochondria-SR ultrastructure. Orange lines are the mitochondria/SR/T-tubulecomplex. Zoomed images highlight the junctions among T-tubule (TT, green), SR (yellow), and mitochondria (Mito, Red). Scale bars: 1 μm (two left images), 500 nm (two right upper images), and 200 nm (two right bottom images). (**B**) Ratio of mitochondria-SR contact length/mitochondrial circumference in cardiomyocytes from sh-scramble and sh-PTPIP51 hearts. (**C**) Mitochondria-SR junction distance in cardiomyocytes from sh-scramble and sh-PTPIP51 hearts. (**D**) Numbers of mitochondria volume density in cardiomyocytes from sh-scramble and sh-PTPIP51 hearts. (**E**) Numbers of tethered mitochondria in 100 randomly selected mitochondria in cardiomyocytes from sh-scramble and sh-PTPIP51 hearts. n = 16, 18 frames in each group from four pairs of mouse hearts. ^*^P < 0.05 versus scramble hearts. (**F**) Schematic view of PTPIP51 regulation of cardiac ischemia/reperfusion through mediating the mitochondria-SR junction.

Moreover, we measured mitochondria-SR junction in PTPIP51 knockdown hearts. Similar with measurement in isolated cardiomyocytes, electron microscopy analysis of PTPIP51 knockdown hearts revealed decreased mitochondria-SR contact (Fig. 6A). The contact ratio of mitochondria-SR membrane in PTPIP51 knockdown hearts was decreased to half of that in scramble control hearts (Fig. 6B). And consistent with results from neonatal cardiomyocytes, PTPIP51 knockdown in the heart widened the distance between mitochondria and SR, from about 35 nm in scramble control hearts to about 50 nm in PTPIP51 knockdown hearts (Fig. 6C). In addition, although mitochondria volume density did not alter in scramble and PTPIP51 knockdown hearts (Fig. 6D), the number of mitochondria that contact SR reduced from 34 per 100 randomly selected mitochondria in scramble control hearts to 23 per 100 mitochondria in PTPIP51 knockdown hearts (Fig. 6E). Thus, our results from both isolated cardiomyocytes and heart tissues confirm the regulatory role of PTPIP51 on mitochondria-SR contact sites.

## Discussion

We have demonstrated a pivotal role of PTPIP51 in regulating cardiac function during I/R through mediating mitochondria-SR contact sites and subsequently mitochondrial calcium and cell fate. PTPIP51 protein was upregulated in I/R hearts. Upregulated PTPIP51 increased the interaction between mitochondria and SR, which in turn caused overload of mitochondrial Ca^2+^ and eventually led to cardiomyocyte death. Knockdown of PTPIP51 or blockage of mitochondrial calcium flux by inhibiting MCU ameliorated H_2_O_2_- or PTPIP51 overexpression-induced cardiomyocyte death. Most importantly, *in vivo* knockdown of PTPIP51 dramatically protected the heart from I/R injury (Fig. 6F).

PTPIP51 is expressed in multiple tissues including epithelial, skeletal muscle, nervous system, adipose, and cancer. Its biochemical structure and possible interacting proteins have been identified^22^,^38^. For instance, the expression of PTPIP51 was observed in human cancer cell lines such as liver carcinoma HepG2 and breast adenocarcinoma MCF7, however, whether there is a causal relationship between PTPIP51 level and carcinogenesis is not clear^23^,^38^. In neurons and axons, PIPIP51 was predicted to play a role in axonal transport through mediating microtubular association based on its structural analysis^23^,^39^,^40^,^41^. More recently, PTPIP51 has been implicated in the pathogenesis of amyotrophic lateral sclerosis, a progressive motor neuron degenerative disease, and interaction of PTPIP51 with VAPB was interrupted by TDP-43, a protein closely associated with amyotrophic lateral sclerosis^30^. In our present study, we found that PTPIP51 expression was upregulated in I/R mouse hearts, suggesting a possible role of PTPIP51 in regulating ischemia-associated heart diseases. Indeed, we further demonstrated that *in vitro* knockdown of PTPIP51 in cardiomyocytes or *in vivo* knockdown in the heart protected against H_2_O_2_-induced cardiomyocyte apoptosis or I/R-induced cardiac injury. Thus, for the first time, our findings provide direct evidence for the pathological function of PTPIP51 in the heart. The mechanism of underlying increased PTPIP51 expression in I/R heart, however, merits further investigation. One speculation is that alterations of cytokines during cardiac I/R, such as MAPK or NFκB pathway-related factors^42^,^43^,^44^,^45^, may contribute to increased PTPIP51 expression. Indeed, *in vitro* studies in cell lines showed that ciliaryneurotrophic factor, leukemia inhibitory factor, or RelA/IκBα negatively regulate expression of PTPIP51^46^,^47^. Moreover, cytokines involved in cardiac I/R such as MAPK and NFκB are also important players in ischemia conditioning protection. Whether regulating PTPIP51 protein level accounts for the conditioning cardioprotective effect is not clear, however, a better understanding of the regulatory mechanism of PTPIP51 expression may help to develop PTPIP51-related cardioprotective strategies.

Intracellular Ca^2+^ homeostasis is necessary for the maintenance of cardiac functions including contraction/relaxation and cell metabolism, and disturbance of Ca^2+^ homeostasis is usually associated with cardiac I/R-related injury^3^,^35^. During cardiac I/R, excessive Ca^2+^ enters the mitochondrial matrix through mitochondrial Ca^2+^ uniporter MCU, which subsequently triggers the mPTP opening, causes excessive ROS production, and eventually leads to cardiomyocyte death and cardiac dysfunction^11^,^32^. We previously found that PTPIP51 primarily localizes on the mitochondrial surface through its N-terminal putative transmembrane motif and causes apoptosis in HEK293T and HeLa cells^23^. The present study further investigated the mechanisms underlying PTPIP51′s apoptotic effects in cardiomyocytes. We found that PTPIP51 caused increased basic mitochondrial Ca^2+^ and Ca^2+^ in response to SR Ca^2+^ release, which could be blocked by either pharmacological inhibition of MCU or molecular knockdown of MCU (Figs 3 and 4). More importantly, inhibition of MCU ameliorated PTPIP51-induced cardiomyocyte apoptosis (Fig. 4). Thus, the present study provides evidence showing that PTPIP51 serves as a novel protein target and contributes to the mitochondrial Ca^2+^ overload and disturbance of intracellular Ca^2+^ homeostasis during cardiac I/R.

Mitochondria and ER are organelles that are in physical contact with each other, and this contact allows lipid and calcium exchange between both organelles^48^. Increasing evidence shows that the transfer of Ca^2+^ from ER to mitochondria through mitochondria-ER junction sites plays a pivotal role in the regulation of mitochondria-dependent apoptosis^13^. For instance, the interaction between the ER protein B-cell receptor-associated protein 31 and the mitochondrial protein FIS1 facilitates the progression of cell apoptosis through triggering Ca^2+^ release from ER to mitochondria^49^. Recently, the central role of mitochondria-ER in multiple diseases such as neurodegenerative diseases and metabolic diseases has been recognized^50^,^51^. In Alzheimer disease, mutants of presenilins and amyloid precursors or protein were found to increase mitochondria-ER contact and upregulate mitochondria-ER function, and the alteration of the mitochondria-ER connection can explain phenotypes associated with Alzheimer disease^52^. More recently, chronic enrichment of hepatic mitochondria-ER coupling has been reported to lead to mitochondrial dysfunction in obesity^53^. Here, our study found that in cardiac I/R, increased PTPIP51 positively regulates mitochondria-SR contact, which in turn increases the uptake of Ca^2+^ into mitochondria, leading to mitochondrial Ca^2+^ overload and cell death. Consistent with our findings in the heart, interaction of PTPIP51 with ER protein VAPB was implicated in the pathogenesis of ALS, the neurodegenerative disease with mitochondrial Ca^2+^ overload as one of the hallmarks^24^,^54^. The mechanism of how PTPIP51 tethers mitochondria with SR is still an open question and needs further investigation. The most likely possibility is that PTPIP51 on the outer mitochondrial membrane directly interacts with adaptor proteins such as VAPB or oxysterol-binding protein-related protein ORP5/ORP8 on SR, facilitating the tethering of these two organelles^24^,^48^. The homo-interaction between PTPIP51 on mitochondria and PTPIP51 on SR could also explain increased mitochondria-SR contact because PTPIP51 was also observed localizing at the ER and in the nucleus^22^.

In summary, our study found that cardiac I/R increased PTPIP51 expression, upregulated PTPIP51 protein caused cardiac injury through enhancing mitochondria-SR contact, increasing Ca^2+^ transfer from SR to mitochondria, causing mitochondrial Ca^2+^ overload and cardiomyocyte death. Knockdown of PTPIP51 dramatically protected the heart from I/R injury and improved cardiac function. Thus, this study reveals the role of PTPIP51 in the heart for the first time, and provides a new therapeutic target for cardiac I/R-related diseases.

## Methods

### Mouse cardiac I/R model

All procedures of animal handling were performed in accordance with animal use guidelines and approved protocols by the Institutional Animal Care and Use Committee of Peking University Health Science Center. Mouse cardiac I/R models were established using male C57BL/6 mice at the age of 8 weeks and following the protocol described previously^55^. Briefly, mice were mildly anesthetized by intraperitoneal injection of pentobarbital sodium (40 mg/kg). The fourth intercostal space over the left chest of the mouse was exposed and the heart was rapidly squeezed out of the thoracic space. The left main descending coronary artery at a site of about 3 mm from its origin was sutured and tied with a slip knot. The heart was then put back in the thoracic space and air was removed by pressing the thoracic wall to prevent pneumothorax. Thirty minutes after ischemia, the slip knot was released by pulling the outside end of the suture smoothly to re-perfuse the myocardium. 1, 6, 12, and 24-h after reperfusion, the ventricular myocardium was harvested and prepared for experiments. Sham-operated mice underwent a similar procedure except for suture tying.

### Isolation and culture of rat neonatal cardiomyocytes

Isolation and culture of neonatal rat cardiomyocytes were performed as previously described^56^. Briefly, Spraque Dawley (postnatal 1–2 days) were euthanized using isoflurane inhalation followed by cervical dislocation. Then ventricles were digested in HBSS solution containing 0.1% trypsin (Invitrogen, USA) and 0.05% type II collagenase (Worthington, USA). Cells were pre-plated for 2 h and the supernatant containing purified cardiomyocytes was collected and cultured for another 48–72 h before transfecting the adenovirus.

### Generation of adenovirus

The full-length cDNA of PTPIP51 (NM_001304802.1) was amplified by PCR with primers: 5′-CACCATGTCTAGACTGGGAGCCCT-3′ (forward) and 5′-TTAGTCTCGTAAAATGACTTCCAG-3′ (reverse), the amplified product was then inserted into pENTR/TEV/D-TOPO vector (Invitrogen). The newly constructed product was recombined with pAd/CMV/V5-DEST vector (Invitrogen). Target sequences for PTPIP51-shRNA was 5′-GGACAAAGCCATTGAACTT-3′, for MCU-shRNA was 5′-GCAAGGAGTTTCTTTCTCTTT-3′, and for MFN2-shRNA was 5′-GGACCCAGTTACTACAGAAGA-3′. shRNA sequences were inserted into pENTR™/U6 vector and then recombined with pBLOCK-iT™ 6-DEST vector. Adenovirus was produced with Adenoviral Expression System (Invitrogen) and purified using Vivapure^®^AdenoPACK^TM^20RT Kit (Sartorius).

### Generation of mouse PTPIP51 knockdown model

PTPIP51 shRNA was inserted into AAV9-ZsGreen, the highly efficient cardiac transfection AAV serotype, to construct the AAV9-ZsGreen-shPTPIP51 virus. 2.0 × 10^11^ plaque-forming units (pfu) of AAV9 (adeno-associated virus serotype 9 vector)-ZsGreen-shScramble and AAV9-ZsGreen-shPTPIP51 (designed and produced by Likeli, Beijing, China) were injected intravenously into 8-week-old C57BL/6 male mice. One month after virus injection, confocal and western blot of heart tissue were performed to confirm the knockdown efficiency.

### Heart echocardiography

Heart transthoracic echocardiography was committed before and after cardiac I/R surgery. Briefly, mice were anesthetized by intraperitoneal injection of pentobarbital sodium (40 mg/kg), then imaged in the left lateral decubitus position with a Vevo710 RMV-707B (Visualsonics) machine. 2-dimensional images were recorded in parasternal long- and short- axis projections, with guided M-mode recordings at the midventricular level. 3 beats from each projection were measured and averaged.

### Immunoblotting

Protein was extracted from mouse myocardium or cultured cardiomyocytes, totally 40–100 μg protein were separated by SDS-PAGE, and transferred to PVDF membranes. Membranes were probed with indicated antibodies (anti-PTPIP51 from Abclonal, anti-α tubulin from Protein tech, anti-MCU from CST) overnight at 4 °C, and then incubated with secondary antibodies (IRDye-conjugated anti-mouse and anti-rabbit IgG from LI-COR) for 2 hours at room temperature. Immunoblots were evaluated using the Odyssey imaging system.

### Cell death assay

Neonatal cardiomyocytes were plated at a density of 5 × 10^4^cells/well in 6-well plates and transfected with adeno-PTPIP51 or adeno-lacZ, or with adeno-PTPIP51 shRNA or adeno-scramble in the presence of 200 μM H_2_O_2_for 12 h. Cells were harvested 48 h after adenovirus transfection and fixed with 4% paraformaldehyde. Cell apoptosis was determined by flow cytometry using Annexin-V-FITC Apoptosis Detection Kit (Dojindo Laboratories, Japan)by flow cytometry analysis, or determined by TUNEL assay using In situ cell death detection kit (Roche, USA),following the manufacture’s instruction.

### Confocal imaging of mitochondria and SR

Cardiomyocytes were loaded with Mito-Tracker Green (0.2 μmol/L, 10 min) and ER-Tracker Red (1 μmol/L, 30 min) (Invitrogen). Confocal imaging was carried out with Zeiss LSM 710 confocal microscope equipped with a 40×, 1.3NA oil immersion objective. Dual excitation imaging of Mito-Tracker Green and ER-Tracker Red were achieved by excitation at 488 and 561 nm, and the emission was collected at 505–530 and >560 nm respectively. Images were analyzed using ImageJ plug-in intensity correlation analysis and Pearson correlation coefficient with blinded analyser^57^,^58^.

### Immunostaining

Neonatal rat cardiomyocytes were fixed in 4% paraformaldehyde at room temperature for 1 h, permeabilized with 0.2% Triton X-100 for 5 min, and blocked in PBS with 5% bovine serum albumin at RT for 30 min. Samples were incubated with indicated antibodies (Mouse-anti-Tom20 from Abcam and Rabbit-anti-PTPIP51 from Abclonal) at 4 °C overnight, washed in PBS, and then incubated with secondary antibodies at 37 °C for 2 h. Imaging of PTPIP51 and Tom20 were acquired by excitation at 488 and 543 nm, and the emissions were collected at 505–530 and >560 nm, respectively.

### Measurement of cellular calcium signals

Cardiomyocytes were loaded with cytosolic calcium probe Fluo-4 AM (5 μmol/L) or mitochondrial calcium probe Rhod-2 AM (5 μmol/L) at 37 °C for 15 min, then washed with Tyrode’s solution consisting of (in mmol/L) 137 NaCl, 5.4KCl, 1.2 MgCl_2_, 1.2 NaH_2_PO4, 10 D-glucose, and 20 HEPES (pH 7.35–7.40, adjusted with NaOH). The Fluo-4 or Rhod-2 fluorescent signals were monitored by Zeiss LSM 710 confocal microscope. For Fluo-4 fluorescence, images were acquired by exciting at 488 nm and collecting the emission at 505–530 nm. 400 frames of 128 × 128 pixels were collected at 0.244 s/frame in a bidirectional scanning mode when cells were treated with caffeine; 200 frames of 512 × 512 pixels were collected at 1.94 s/frame when treated with FCCP. For Rhod-2 fluorescence, images were taken by exciting at 543 nm and collecting the emission at >560 nm. 200 frames of 512 × 512 pixels were collected at 1.94 s/frame in a bidirectional scanning mode.

### Transmission electron microscopy analyses

Cardiomyocytes were fixed with 2% glutaraldehyde in sodium cacodylate buffer (0.1 mol/L, pH 7.2) at 4 °C overnight and post-fixed for 1 h in 1% osmium tetroxide. Cells were then stained for 1 h with 1% uranyl acetate in water before dehydration and embedding in TAAB resin. Digital images were acquired by a JEM-1230 High Contrast Transmission Electron Microscope and Soft Imaging system (JEOL, Japan). The circumference of each mitochondrion and the proportions of the mitochondrial surface closely associated with SR (<30 nm, distance for smooth SR-mitochondria with efficient calcium exchange)^59^ were calculated at high resolution (40,000–80,000×) obtained from PTPIP51 cDNA or shRNA transfected neonatal cardiac myocytes and the left ventricle myocardium of AAV9 sh-Scramble or AAV9 sh-PTPIP51-transfected mice. All clearly identified mitochondria were scored. To obtain an estimate of the minimum distance between sites of SR release and mitochondrial Ca^2+^ uptake, 5 random points from SR to the nearest mitochondrial outer membrane were drawn with straight lines and measured. Image analysis was performed using Image Pro Plus 6.0 software (Media CybernerticsInc., USA).

### Calculation of mitochondrial tethering and mitochondrial numbers

Mean tether and mitochondria density were measured from electron micrographs taken at 15,000–20,000x magnification. The numbers of tethers (T-Tubule/SR/Mitochondria macrocomplex) and mitochondria were measured from 18 to 20 random non-overlapping regions that were randomly collected from four pairs of AAV9 sh-scramble and AAV9 sh-PTPIP51-transfected mice. Mitochondria interacted points were counted in a 19 * 14 points lattice on 11.9 × 8.6 μm (15000X) EM image, and the volume density was defined as the ratio of interacted points to total points (N/266). The results presented were mean (±SD) number of tethers/100mitochondria and mitochondrial volume density. Image analyses were performed using Image Pro Plus 6.0 software.

### Reagents

Fluo-4 AM, rhod-2 AM, Mito-Tracker, and ER-Tracker were from Invitrogen. Caffeine and Ru360 were from Millipore, FCCP from Sigma.

### Statistical analysis

Data are presented as mean ± SEM. Statistical significance of differences between groups was analyzed by unpaired two-tailed Student’s t-test or one-way ANOVA followed by Bonferroni when more than two groups were compared, and nested ANOVA when cellular observations from several animals. P < 0.05 was considered statistically significant.

## Additional Information

**How to cite this article:** Qiao, X. *et al*. PTPIP51 regulates mouse cardiac ischemia/reperfusion through mediating the mitochondria-SR junction. *Sci. Rep.* **7**, 45379; doi: 10.1038/srep45379 (2017).

**Publisher's note:** Springer Nature remains neutral with regard to jurisdictional claims in published maps and institutional affiliations.

## Supplementary Material

Supplementary Figures

## References

[b1] Mozaffarian D. . Heart disease and stroke statistics–2015 update: A report from the American Heart Association. Circulation. 131, e29–e322 (2015). 25520374 10.1161/CIR.0000000000000152

[b2] Ferdinandy P., Schulz R. & Baxter G. F. Interaction of cardiovascular risk factors with myocardial ischemia/reperfusion injury, preconditioning, and postconditioning. Pharmacol Rev. 59, 418–458 (2007). 18048761 10.1124/pr.107.06002

[b3] Hausenloy D. J. & Yellon D. M. Myocardial ischemia-reperfusion injury: A neglected therapeutic target. J Clin Invest. 123, 92–100 (2013). 23281415 10.1172/JCI62874 PMC3533275

[b4] Prasad A., Stone G. W., Holmes D. R. & Gersh B. Reperfusion injury, microvascular dysfunction, and cardioprotection: The “dark side” of reperfusion. Circulation. 120, 2105–2112 (2009). 19933949 10.1161/CIRCULATIONAHA.108.814640

[b5] Kalogeris T., Baines C. P., Krenz M. & Korthuis R. J. Cell biology of ischemia/reperfusion injury. Int Rev Cell Mol Biol. 298, 229–317 (2012). 22878108 10.1016/B978-0-12-394309-5.00006-7 PMC3904795

[b6] Mozaffari M. S., Liu J. Y., Abebe W. & Baban B. Mechanisms of load dependency of myocardial ischemia reperfusion injury. Am J Cardiovasc Dis. 3, 180–196 (2013). 24224132 PMC3819580

[b7] Liu Y., Sato T., O’Rourke B. & Marban E. Mitochondrial ATP-dependent potassium channels: Novel effectors of cardioprotection? Circulation. 97, 2463–2469 (1998). 9641699 10.1161/01.cir.97.24.2463

[b8] Friedman J. R. & Nunnari J. Mitochondrial form and function. Nature. 505, 335–343 (2014). 24429632 10.1038/nature12985 PMC4075653

[b9] Esposito L. A., Melov S., Panov A., Cottrell B. A. & Wallace D. C. Mitochondrial disease in mouse results in increased oxidative stress. Proc Natl Acad Sci USA. 96, 4820–4825 (1999). 10220377 10.1073/pnas.96.9.4820 PMC21775

[b10] Perrelli M. G., Pagliaro P. & Penna C. Ischemia/reperfusion injury and cardioprotective mechanisms: Role of mitochondria and reactive oxygen species. World J Cardiol. 3, 186–200 (2011). 21772945 10.4330/wjc.v3.i6.186 PMC3139040

[b11] Honda H. M., Korge P. & Weiss J. N. Mitochondria and ischemia/reperfusion injury. Ann N Y Acad Sci. 1047, 248–258 (2005). 16093501 10.1196/annals.1341.022

[b12] Murphy E. & Steenbergen C. Mechanisms underlying acute protection from cardiac ischemia-reperfusion injury. Physiol Rev. 88, 581–609 (2008). 18391174 10.1152/physrev.00024.2007 PMC3199571

[b13] Rowland A. A. & Voeltz G. K. Endoplasmic reticulum-mitochondria contacts: Function of the junction. Nat Rev Mol Cell Biol. 13, 607–625 (2012). 22992592 10.1038/nrm3440 PMC5111635

[b14] Rizzuto R. . Close contacts with the endoplasmic reticulum as determinants of mitochondrial Ca2+ responses. Science. 280, 1763–1766 (1998). 9624056 10.1126/science.280.5370.1763

[b15] Boncompagni S. . Mitochondria are linked to calcium stores in striated muscle by developmentally regulated tethering structures. Mol Biol Cell. 20, 1058–1067 (2009). 19037102 10.1091/mbc.E08-07-0783 PMC2633377

[b16] Aurora A. B. . MicroRNA-214 protects the mouse heart from ischemic injury by controlling Ca(2)(+) overload and cell death. J Clin Invest. 122, 1222–1232 (2012). 22426211 10.1172/JCI59327 PMC3314458

[b17] Dorn G. N. & Scorrano L. Two close, too close: Sarcoplasmic reticulum-mitochondrial crosstalk and cardiomyocyte fate. Circ Res. 107, 689–699 (2010). 20847324 10.1161/CIRCRESAHA.110.225714 PMC2963937

[b18] Szabadkai G. . Chaperone-mediated coupling of endoplasmic reticulum and mitochondrial Ca2+ channels. J Cell Biol. 175, 901–911 (2006). 17178908 10.1083/jcb.200608073 PMC2064700

[b19] de Brito O. M. & Scorrano L. Mitofusin 2 tethers endoplasmic reticulum to mitochondria. Nature. 456, 605–610 (2008). 19052620 10.1038/nature07534

[b20] Chen Y. . Mitofusin 2-Containing Mitochondrial-Reticular microdomains direct rapid cardiomyocyte bioenergetic responses via interorganelle ca2+ crosstalk. Circ Res. 111, 863–875 (2012). 22777004 10.1161/CIRCRESAHA.112.266585 PMC3444672

[b21] Paillard M. . Depressing mitochondria-reticulum interactions protects cardiomyocytes from lethal hypoxia-reoxygenation injury. Circulation. 128, 1555–1565 (2013). 23983249 10.1161/CIRCULATIONAHA.113.001225

[b22] Stenzinger A., Schreiner D., Koch P., Hofer H. W. & Wimmer M. Cell and molecular biology of the novel protein tyrosine-phosphatase-interacting protein 51. Int Rev Cell Mol Biol. 275, 183–246 (2009). 19491056 10.1016/S1937-6448(09)75006-3

[b23] Lv B. F. . Protein tyrosine phosphatase interacting protein 51 (PTPIP51) is a novel mitochondria protein with an N-terminal mitochondrial targeting sequence and induces apoptosis. Apoptosis. 11, 1489–1501 (2006). 16820967 10.1007/s10495-006-8882-9

[b24] De Vos K. J. . VAPB interacts with the mitochondrial protein PTPIP51 to regulate calcium homeostasis. Hum Mol Genet. 21, 1299–1311 (2012). 22131369 10.1093/hmg/ddr559 PMC3284118

[b25] Morotz G. M. . Amyotrophic lateral sclerosis-associated mutant VAPBP56S perturbs calcium homeostasis to disrupt axonal transport of mitochondria. Hum Mol Genet. 21, 1979–1988 (2012). 22258555 10.1093/hmg/dds011 PMC3315205

[b26] Stoica R. . ALS/FTD-associated FUS activates GSK-3beta to disrupt the VAPB-PTPIP51 interaction and ER-mitochondria associations. Embo Rep. (2016). 10.15252/embr.201541726 PMC5007559 27418313

[b27] Hernandez Y. J. . Latent adeno-associated virus infection elicits humoral but not cell-mediated immune responses in a nonhuman primate model. J Virol. 73, 8549–8558 (1999). 10482608 10.1128/jvi.73.10.8549-8558.1999 PMC112875

[b28] Zincarelli C., Soltys S., Rengo G. & Rabinowitz J. E. Analysis of AAV serotypes 1-9 mediated gene expression and tropism in mice after systemic injection. Mol Ther. 16, 1073–1080 (2008). 18414476 10.1038/mt.2008.76

[b29] Hajjar R. J. Potential of gene therapy as a treatment for heart failure. J Clin Invest. 123, 53–61 (2013). 23281410 10.1172/JCI62837 PMC3533270

[b30] Stoica R. . ER-mitochondria associations are regulated by the VAPB-PTPIP51 interaction and are disrupted by ALS/FTD-associated TDP-43. Nat Commun. 5, 3996 (2014). 24893131 10.1038/ncomms4996 PMC4046113

[b31] Elrod J. W. . Cyclophilin D controls mitochondrial pore-dependent Ca(2+) exchange, metabolic flexibility, and propensity for heart failure in mice. J Clin Invest. 120, 3680–3687 (2010). 20890047 10.1172/JCI43171 PMC2947235

[b32] Giorgi C. . Mitochondrial Ca(2+) and apoptosis. Cell Calcium. 52, 36–43 (2012). 22480931 10.1016/j.ceca.2012.02.008 PMC3396846

[b33] Kirichok Y., Krapivinsky G. & Clapham D. E. The mitochondrial calcium uniporter is a highly selective ion channel. Nature. 427, 360–364 (2004). 14737170 10.1038/nature02246

[b34] Luongo T. S. . The mitochondrial calcium uniporter matches energetic supply with cardiac workload during stress and modulates permeability transition. Cell Rep. 12, 23–34 (2015). 26119731 10.1016/j.celrep.2015.06.017 PMC4517182

[b35] Rizzuto R., De Stefani D., Raffaello A. & Mammucari C. Mitochondria as sensors and regulators of calcium signalling. Nat Rev Mol Cell Biol. 13, 566–578 (2012). 22850819 10.1038/nrm3412

[b36] Csordas G. . Structural and functional features and significance of the physical linkage between ER and mitochondria. J Cell Biol. 174, 915–921 (2006). 16982799 10.1083/jcb.200604016 PMC2064383

[b37] Li Q. . A syntaxin 1, Galpha(o), and N-type calcium channel complex at a presynaptic nerve terminal: Analysis by quantitative immunocolocalization. J Neurosci. 24, 4070–4081 (2004). 15102922 10.1523/JNEUROSCI.0346-04.2004 PMC6729428

[b38] Stenzinger A. . The novel protein PTPIP51 exhibits tissue- and cell-specific expression. Histochem Cell Biol. 123, 19–28 (2005). 15609043 10.1007/s00418-004-0732-7

[b39] Gainer H. & Chin H. Molecular diversity in neurosecretion: Reflections on the hypothalamo-neurohypophysial system. Cell Mol Neurobiol. 18, 211–230 (1998). 9535291 10.1023/A:1022568904002 PMC11560193

[b40] Senda T. & Yu W. Kinesin cross-bridges between neurosecretory granules and microtubules in the mouse neurohypophysis. Neurosci Lett. 262, 69–71 (1999). 10076875 10.1016/s0304-3940(99)00042-7

[b41] Oishi K., Okano H. & Sawa H. RMD-1, a novel microtubule-associated protein, functions in chromosome segregation in Caenorhabditis elegans. J Cell Biol. 179, 1149–1162 (2007). 18070910 10.1083/jcb.200705108 PMC2140014

[b42] Zhang X. Q. . Cardiomyocyte-specific p65 NF-kappaB deletion protects the injured heart by preservation of calcium handling. Am J Physiol Heart Circ Physiol. 305, H1089–H1097 (2013). 23913709 10.1152/ajpheart.00067.2013 PMC3798748

[b43] Liu Y. . Inhibiting (pro)renin receptor-mediated p38 MAPK signaling decreases hypoxia/reoxygenation-induced apoptosis in H9c2 cells. Mol Cell Biochem. 403, 267–276 (2015). 25711402 10.1007/s11010-015-2356-8

[b44] Onai Y. . Inhibition of IkappaB phosphorylation in cardiomyocytes attenuates myocardial ischemia/reperfusion injury. Cardiovasc Res. 63, 51–59 (2004). 15194461 10.1016/j.cardiores.2004.03.002

[b45] Heidbreder M., Naumann A., Tempel K., Dominiak P. & Dendorfer A. Remote vs. Ischaemic preconditioning: The differential role of mitogen-activated protein kinase pathways. Cardiovasc Res. 78, 108–115 (2008). 18096574 10.1093/cvr/cvm114

[b46] Roger J., Goureau O., Sahel J. A. & Guillonneau X. Use of suppression subtractive hybridization to identify genes regulated by ciliary neurotrophic factor in postnatal retinal explants. Mol Vis. 13, 206–219 (2007). 17327826 PMC2610405

[b47] Brobeil A. . PTPIP51-A new RelA-tionship with the NFkappaB signaling pathway. Biomolecules. 5, 485–504 (2015). 25893721 10.3390/biom5020485 PMC4496682

[b48] Galmes R. . ORP5/ORP8 localize to endoplasmic reticulum-mitochondria contacts and are involved in mitochondrial function. Embo Rep. 17, 800–810 (2016). 27113756 10.15252/embr.201541108 PMC5278607

[b49] Iwasawa R., Mahul-Mellier A. L., Datler C., Pazarentzos E. & Grimm S. Fis1 and Bap31 bridge the mitochondria-ER interface to establish a platform for apoptosis induction. Embo J. 30, 556–568 (2011). 21183955 10.1038/emboj.2010.346 PMC3034017

[b50] Lopez-Crisosto C. . ER-to-mitochondria miscommunication and metabolic diseases. Biochim Biophys Acta. 1852, 2096-2105 (2015). 10.1016/j.bbadis.2015.07.011 26171812

[b51] Cali T., Ottolini D. & Brini M. Calcium and endoplasmic reticulum-mitochondria tethering in neurodegeneration. Dna Cell Biol. 32, 140–146 (2013). 23477673 10.1089/dna.2013.2011

[b52] Area-Gomez E. . Upregulated function of mitochondria-associated ER membranes in Alzheimer disease. Embo J. 31, 4106–4123 (2012). 22892566 10.1038/emboj.2012.202 PMC3492725

[b53] Arruda A. P. . Chronic enrichment of hepatic endoplasmic reticulum-mitochondria contact leads to mitochondrial dysfunction in obesity. Nat Med. 20, 1427–1435 (2014). 25419710 10.1038/nm.3735 PMC4412031

[b54] Bergmann F. & Keller B. U. Impact of mitochondrial inhibition on excitability and cytosolic Ca2+ levels in brainstem motoneurones from mouse. J Physiol. 555, 45–59 (2004). 14660707 10.1113/jphysiol.2003.053900 PMC1664822

[b55] Gao E. . A novel and efficient model of coronary artery ligation and myocardial infarction in the mouse. Circ Res. 107, 1445–1453 (2010). 20966393 10.1161/CIRCRESAHA.110.223925 PMC3005817

[b56] Tanaka M. . Hypoxia induces apoptosis with enhanced expression of Fas antigen messenger RNA in cultured neonatal rat cardiomyocytes. Circ Res. 75, 426–433 (1994). 7520371 10.1161/01.res.75.3.426

[b57] Worz S. . 3D Geometry-Based quantification of colocalizations in multichannel 3D microscopy images of human soft tissue tumors. Ieee T Med Imaging. 29, 1474–1484 (2010). 10.1109/TMI.2010.2049857 20562043

[b58] Reitan N. K., Sporsheim B., Bjorkoy A., Strand S. & Davies C. L. Quantitative 3-D colocalization analysis as a tool to study the intracellular trafficking and dissociation of pDNA-chitosan polyplexes. J Biomed Opt. 17, 26015 (2012). 10.1117/1.JBO.17.2.026015 22463047

[b59] Giacomello M. & Pellegrini L. The coming of age of the mitochondria–ER contact: A matter of thickness. Cell Death Differ. 23, 1417–1427 (2016). 27341186 10.1038/cdd.2016.52 PMC5072433

