# Supplementary material for: PTPIP51 regulates mouse cardiac ischemia/reperfusion through mediating the mitochondria-SR junction
Source: Sci Rep. 2017 Mar 27;7:45379. doi: 10.1038/srep45379 (PMC5366942; doi:10.1038/srep45379)
Supplement: Supplementary Figures [file srep45379-s1.pdf]

PTPIP51 regulates mouse cardiac ischemia/reperfusion through mediating the mitochondria-SR junction

Xue Qiao[1], Shi Jia[1], Jingjing Ye[1], Xuan Fang[1], Chenglin Zhang[1], Yangpo Cao[1], Chunling Xu[1], Lifang Zhao[1],Yi Zhu[1], Lu Wang[2], Ming Zheng[1*]

1) Department of Physiology and Pathophysiology, and 2) Center for Human Disease Genomics, School of Basic Medical Sciences, Health Science Center, Peking University, Beijing 100191, China

Running title: PTPIP51 in Cardiac Ischemia/Reperfusion

Address Correspondence to:
Ming Zheng, M.D., Ph.D.
Department of Physiology and Pathophysiology
Health Science Center, Peking University
Beijing 100191
China
Tel.: 86-10-8280-2403
Fax: 86-10-8280-2403
E-Mail: zhengm@bjmu.edu.cn

Supplemental Figure Legends

Figure S1. Echocardiography of mouse I/R model and knockdown of PTPIP51 protected cardiomyocytes from H_2O_2 mediated apoptosis. A, Representative of M-mode echocardiography of sham or I/R mouse. **B**, Mean data of ejection fraction (EF) and fractional shortening (FS). n=4 pairs of mice. $^{*}P<0.05$ versus sham mouse. **C,** TUNEL staining and **D,** Annexin V-PI by flow cytometry in rat neonatal cardiomyocytes transfected with scramble or PTPIP51 shRNA in the presence of H_2O_2 (200 μM, 12 h).

Figure S2. Generation and characteristics of PTPIP51 knockdown mouse model and its cardiac protective effects after I/R. A, Schematic view of constructing an AAV9-mediated PTPIP51 shRNA mice model. **B,** M-mode echocardiography of AAV9-sh-Scramble and AAV9-sh-PTPIP51 mice before and after I/R. **C,** TUNEL assay of hearts from indicated mice. n=7–14 frames from four pairs of mice. $^{*}P<0.05$ versus sham scramble; $^{\#}P<0.05$ versus scramble with I/R injury.

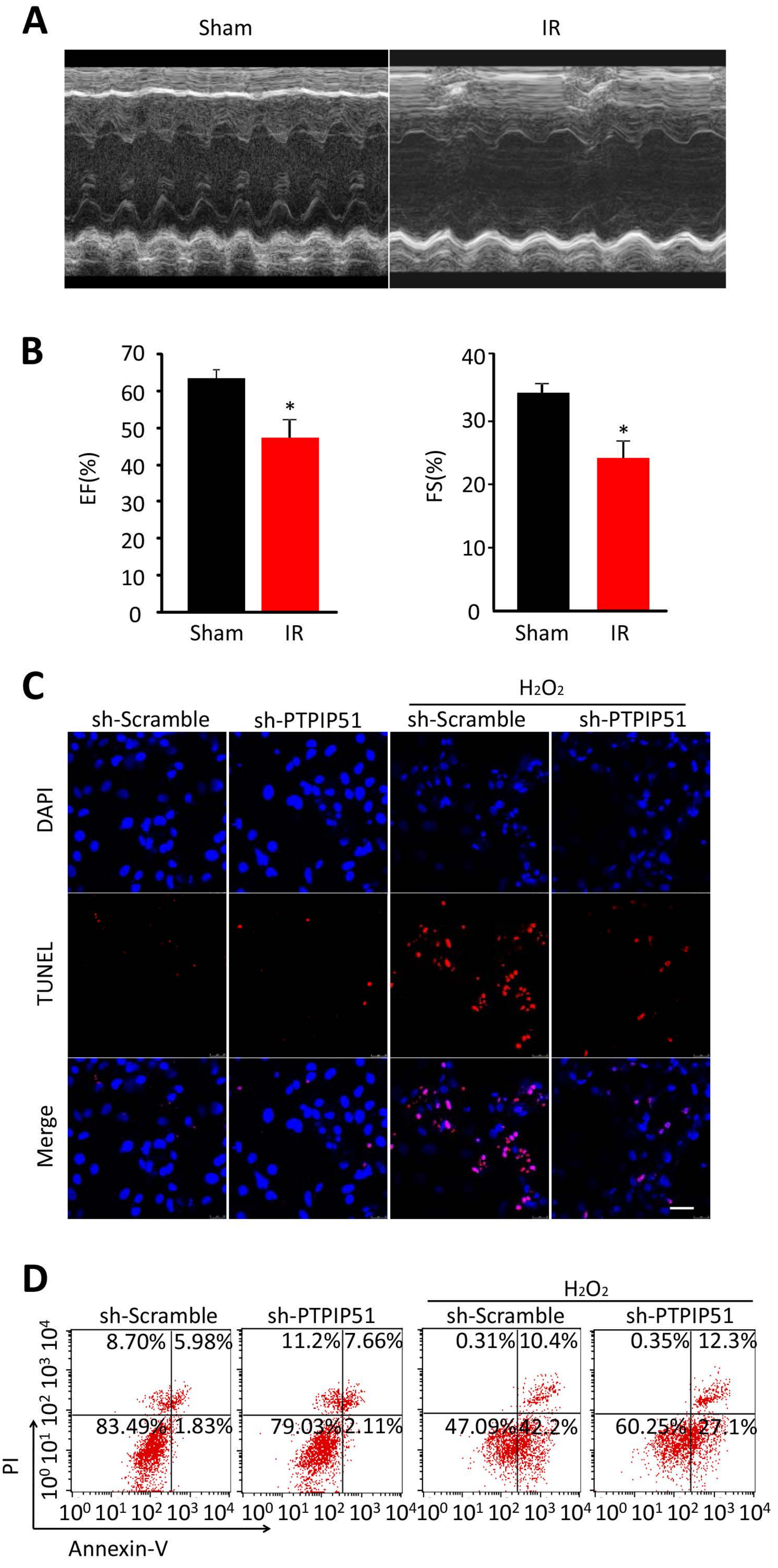

A
Sham
IR
B
EF(%)
70
60
50
40
30
20
10
0
Sham
IR
*
FS(%)
40
30
20
10
0
Sham
IR
*
C
H2O2
sh-Scramble
sh-PTPIP51
sh-Scramble
sh-PTPIP51
DAPI
TUNEL
Merge
D
H2O2
sh-Scramble
sh-PTPIP51
sh-Scramble
sh-PTPIP51
8.70%
5.98%
83.49%
1.83%
11.2%
7.66%
79.03%
2.11%
0.31%
10.4%
47.09%
42.2%
0.35%
12.3%
60.25%
27.1%
PI
Annexin-V

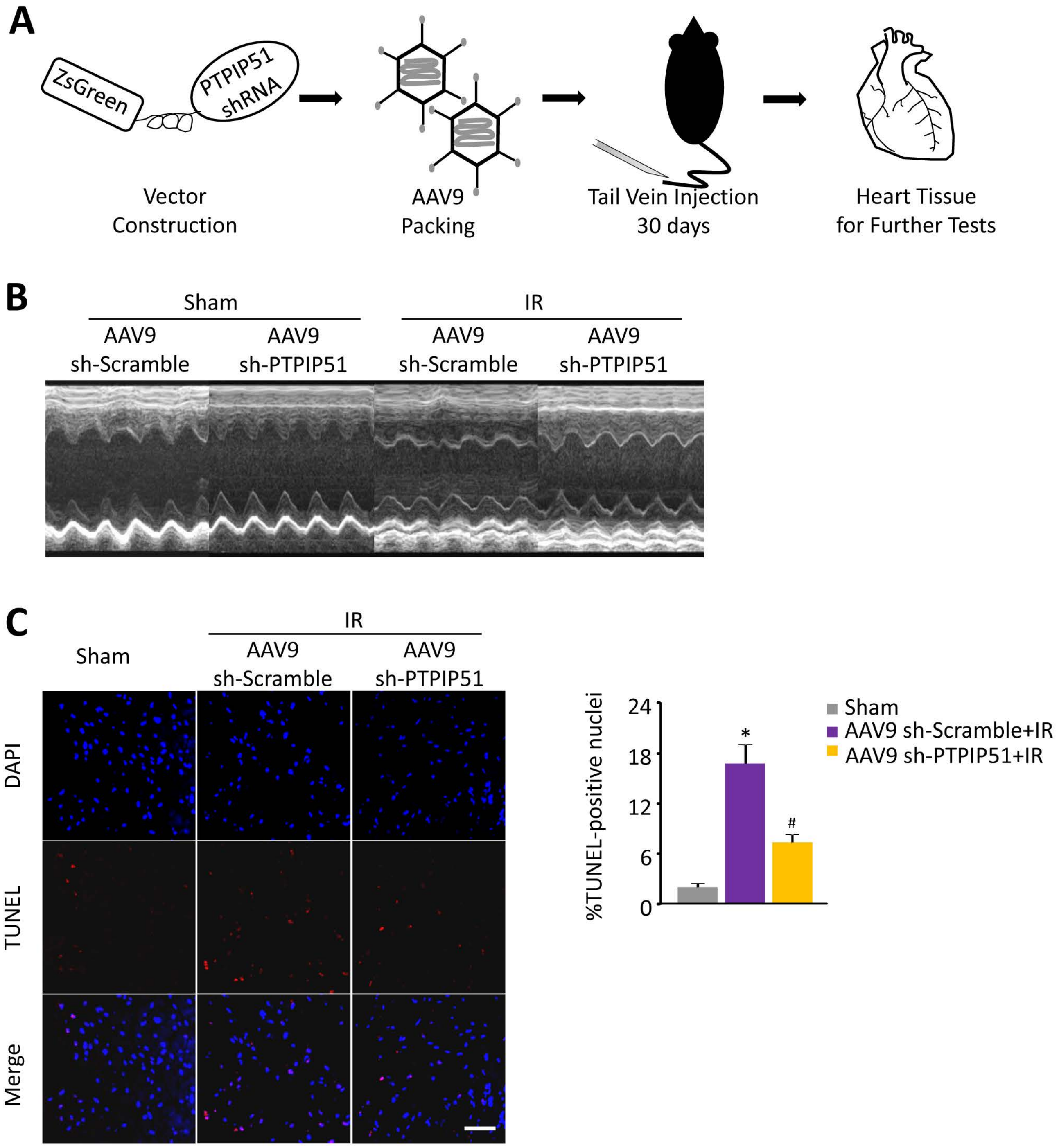

A
ZsGreen
PTPIP51 shRNA
Vector Construction
AAV9 Packing
Tail Vein Injection 30 days
Heart Tissue for Further Tests
B
Sham
IR
AAV9 sh-Scramble
AAV9 sh-PTPIP51
AAV9 sh-Scramble
AAV9 sh-PTPIP51
C
Sham
IR
AAV9 sh-Scramble
AAV9 sh-PTPIP51
DAPI
TUNEL
Merge
%TUNEL-positive nuclei
24
18
12
6
0
*
#
Sham
AAV9 sh-Scramble+IR
AAV9 sh-PTPIP51+IR
